# Dual Roles for Nucleus Accumbens Core Dopamine D1-Expressing Neurons Projecting to the Substantia Nigra Pars Reticulata in Limbic and Motor Control in Male Mice

**DOI:** 10.1523/ENEURO.0082-23.2023

**Published:** 2023-06-05

**Authors:** Suthinee Attachaipanich, Takaaki Ozawa, Tom Macpherson, Takatoshi Hikida

**Affiliations:** Laboratory for Advanced Brain Functions, Institute for Protein Research, Osaka University, Osaka 565-0871, Japan

**Keywords:** basal ganglia, dopamine, motor, nucleus accumbens, reward, substantia nigra

## Abstract

The nucleus accumbens (NAc) is a critical component of a limbic basal ganglia circuit that is thought to play an important role in decision-making and the processing of rewarding stimuli. As part of this circuit, dopamine D1 receptor-expressing medium spiny neurons (D1-MSNs) of the NAc core are known to send a major projection to the substantia nigra pars reticulata (SNr). However, the functional role of this SNr-projecting NAc D1-MSN (NAc^D1-MSN^–SNr) pathway is still largely uncharacterized. Moreover, as the SNr is thought to belong to both limbic and motor information-processing basal ganglia loops, it is possible that the NAc^D1-MSN^–SNr pathway may be able to influence both limbic and motor functions. In this study, we investigated the effect of optogenetic manipulation of the NAc^D1-MSN^–SNr pathway on reward-learning and locomotor behavior in male mice. Stimulation of the axon terminals of NAc core D1-MSNs in the SNr induced a preference for a laser-paired location, self-stimulation via a laser-paired lever, and augmented instrumental responding for a liquid reward-paired lever. Additionally, stimulation was observed to increase locomotor behavior when delivered bilaterally and induced contralateral turning behavior when delivered unilaterally. However, interestingly, inhibition of this pathway did not alter either reward-related behaviors or locomotion. These findings indicate that the NAc^D1-MSN^–SNr pathway is able to control both reward learning and motor behaviors.

## Significance Statement

The nucleus accumbens (NAc) has been implicated in both limbic and motor control; however, the specific cell types and pathways by which this is achieved have been unclear. Here we demonstrate that activity in NAc core dopamine D1 receptor-expressing medium spiny neurons (D1-MSNs) projecting to the substantia nigra pars reticulata (SNr) is able to modulate both reinforcement and forward motion in male mice, providing further evidence for dual roles for the NAc in limbic and motor circuits.

## Introduction

The nucleus accumbens (NAc) is a key component of the reward and motivation system of the brain and is a major input nucleus of the basal ganglia, a group of nuclei that are important for learning and motor control (Floresco, 2015; Salgado and Kaplitt, 2015; Macpherson and Hikida, 2019). Classically, the NAc has been delineated into at least two subregions, the central core (NAc core) and the surrounding shell (NAc shell), based on the expression of various histochemical markers, as well as topographically organized afferent inputs (Záborszky et al., 1985; Jongen‐Rêlo et al., 1994; Groenewegen et al., 1999; Zahm, 1999; Voorn et al., 2004; Ma et al., 2020). Within both the NAc core and shell, medium spiny neurons (MSNs), the major neuron type, are typically subdivided into two subpopulations of approximately equal size according to their expression of dopamine receptors, as follows: dopamine D1 receptor-expressing MSNs (D1-MSNs) and D2-MSNs (Gerfen et al., 1990). While NAc D1-MSNs have been implicated in reward learning and the attribution of motivational salience (Hikida et al., 2010; Lobo et al., 2010; Macpherson et al., 2014; Macpherson and Hikida, 2018), D2-MSNs have been indicated to play an important role in aversive learning, stimulus discrimination, and behavioral flexibility (Hikida et al., 2010; Yawata et al., 2012; Macpherson et al., 2016; Iino et al., 2020; Macpherson et al., 2022; Nishioka et al., 2023). Additionally, it has been reported that NAc D1-MSNs and D2-MSNs may also contribute to the control of locomotor activity. Intra-NAc infusions of both D1 and D2 receptor agonists have been reported to increase locomotion in rats, while intra-NAc infusion of D1 and D2 antagonists reduced locomotor activity (Dreher and Jackson, 1989; Plaznik et al., 1989). In partial agreement with these studies, more recently, chemogenetic activation of NAc D1-MSNs was demonstrated to increase, while activation of NAc D2-MSNs was demonstrated to decrease, wheel running and locomotor activity in an open field arena (Zhu et al., 2016). These studies indicate that NAc D1-MSNs and D2-MSNs may contribute to the functional control of limbic as well as locomotor processing.

In the NAc core, D2-MSNs largely project to the ventral pallidum (VP), while NAc D1-MSNs are known to send equal projections to both the VP (NAc^D1-MSN^–VP) and the substantia nigra pars reticulata [SNr (NAc^D1-MSN^–SNr); Heimer et al., 1991; Robertson and Jian, 1995; Kupchik et al., 2015; Kupchik and Kalivas, 2017; Matsui and Alvarez, 2018; Pardo-Garcia et al., 2019]. While the NAc^D1-MSN^–VP pathway has been established to play an important role in cocaine addiction-related behaviors, including cue-induced reinstatement of cocaine-seeking and cocaine-induced locomotor sensitization (Stefanik et al., 2013; Creed et al., 2016; Pardo-Garcia et al., 2019), little is currently known about the functional role of the NAc^D1-MSN^–SNr pathway. Classically, NAc D1-MSN projections to the SNr have been suggested to form an integral part of a limbic information processing basal ganglia loop circuit (Alexander et al., 1986; Foster et al., 2021; Haber, 2003; Macpherson et al., 2021; Parent and Hazrati, 1995), in which activation of the NAc^D1-MSN^–SNr pathway is hypothesized to drive reinforcement; however, this proposed role is yet to be empirically tested (Balleine, 2019; Macpherson et al., 2021; Peak et al., 2019). Interestingly, recent evidence has indicated that the NAc^D1-MSN^–SNr pathway may also be able to influence motor behavior. Indeed, optogenetic activation of axon terminals in the SNr in NAc D1-MSNs was reported to increase activity not only in the mPFC, but also in the primary motor cortex (M1; Aoki et al., 2019). While this study highlights the potential for the NAc^D1-MSN^–SNr pathway to exert an influence over motor areas such as the M1, it has yet to be established whether activation of this pathway results in changes in motor behavior.

Here we used optogenetic manipulation of the axon terminals of NAc core D1-MSNs in the SNr to investigate the contribution of the NAc^D1-MSN^–SNr pathway to limbic and motor functions. We reveal that stimulation of this NAc-to-SNr pathway was able to induce a strong reinforcing effect in place preference and self-stimulation tasks and could augment instrumental responding for a liquid reward. Further, we demonstrate that optogenetic activation of the NAc^D1-MSN^–SNr pathway resulted in increased motor activity in an open field arena. However, interestingly, inhibition of this pathway did not significantly affect either reward-related behaviors or locomotion. These findings indicate that activity in the NAc^D1-MSN^–SNr pathway is able to play a dual role in augmenting limbic and motor functions and highlights the potential of targeting this circuit for interventions for clinical conditions associated with limbic or motor impairments.

## Materials and Methods

### Animals

Group-housed male Drd1a-Cre (hereafter referred to as D1-Cre) transgenic mice (FK150Gsat, The Jackson Laboratory) 8–12 weeks of age and on a C57BL/6 background, as well as their wild-type counterparts, were used for experiments. Animals were housed in groups of two to three and were maintained on a 12 h light/dark cycle (lights on at 8:00 A.M.) with the temperature controlled to 24 ± 2°C in a humidity of 50 ± 5%. Behavioral experiments were conducted during the light period. Mice were provided access to water and standard laboratory chow *ad libitum*, except for during touchscreen operant chamber experiments during which time mice were food restricted to maintain motivation to instrumentally respond (see subsection Operant chamber tests of reinforcement). All animal experiments conformed to the guidelines of the National Institutes of Health experimental procedures and were approved by the ethical committee of Osaka University.

### Stereotaxic virus injection and optical cannula implantation

Following anesthesia (90 mg/kg ketamine, i.p., and 20 mg/kg xylazine, i.p.), mice were positioned in a stereotaxic apparatus. A midline incision was made down the scalp, and a craniotomy was made using a dental drill. Injections were conducted using graduated pipettes with a tip diameter of 10–15 μm. Mice were bilaterally injected into the NAc core (bregma coordinates: anteroposterior, +1.2 mm; mediolateral, ± 1.35 mm; dorsoventral, −3.75 mm, 250 nl/site at a rate of 100 nl/min) with a Cre-dependent adeno-associated virus (AAV) virus expressing channelrhodopsin-2 [ChR2; AAV2-Ef1a-FLEX-hChR2(H134R)-EYFP; catalog #20298, Addgene] at a concentration of 4.4 × 10^12^ virus molecules/ml, archaerhodopsin (ArchT; AAV5-FLEX-ArchT3.0-tdTomato; catalog #28305, Addgene) at a concentration of 1.3 × 10^13^ virus molecules/ml or an optically inactive control virus (AAV5-Ef1a-DIO-EYFP; catalog #27056, Addgene) at a concentration of 5.3 × 10^12^ virus molecules/ml. All viruses were purchased from Addgene. Following infusion, the needle was kept at the injection site for 10 min to allow for diffusion of the virus and then slowly withdrawn. For robust viral expression, viral infusions occurred a minimum of 3–4 weeks before behavioral training. For behavioral experiments, chronically implantable optic fibers (core, 200 μm; 0.22 numerical aperture; Thorlabs) threaded through ceramic zirconia ferrules were implanted bilaterally into the medial SNr (bregma coordinates: anteroposterior, −3.3 mm; mediolateral, ± 1.0 mm; dorsoventral, −4.5 mm). Finally, three skull screws were implanted 1 mm into the skull surrounding the optic fibers, and the whole skull was secured using dental cement.

Laser stimulation of 473 nm at 20 Hz [for ChR2-expressing or enhanced yellow fluorescent protein (EYFP)-expressing mice] or 532 nm at a constant rate (for ArchT-expressing mice) was delivered by DPSS (diode-pumped solid-state) lasers (Shanghai Laser & Optics Century Co., Ltd.) controlled by a microcontroller (Arduino) at an intensity of 8–10 mW at the fiber tip.

### Behavioral testing

#### Real-time place preference test

The real-time place preference (rt-PP) test was conducted in a white rectangular box divided lengthways down the center into two equal-sized rectangular chambers (width, 15 cm × length, 20 cm × height, 25 cm). Each chamber contained different contextual cues; one chamber had green triangles on the walls, while the other chamber had blue dots on the walls.

The rt-PP task was composed of the following two stages: (1) a preconditioning test (pretest); and (2) test sessions (laser test). During the pretest, mice were able to freely explore the entire apparatus for 15 min, and the time spent in each chamber was measured to check for any bias to either side by using automated video tracking software (EthoVision XT 16, Noldus). Next, the laser test sessions were performed using an unbiased experimental design across 3 consecutive days for 20 min each day. When an animal entered into one chamber, the laser was delivered for the length of time that the animal stayed in the chamber, whereas when the animal entered the other chamber, no stimulation was delivered. The chamber (triangle or dot walls) paired with laser stimulation was randomized between animals. The time spent in the laser-paired chamber was measured across the three test sessions and then compared with the time spent in the non-laser-paired chamber to assess the preference of the mouse.

#### Operant chamber tests of reinforcement

Operant tasks were conducted in trapezoidal Bussey–Saksida touchscreen operant chambers (Lafayette Instrument) housed within a light-atteuating and sound-attenuating cubicle. In each chamber, the front touchscreen was divided into two touch response panels (70 × 75 mm^2^ spaced, 5 mm apart, 16 mm above the floor), and a liquid delivery magazine was placed at the back end of the chamber. Self-stimulation tests were controlled by ABET II and Whisker Server software (Lafayette Instrument), and laser delivery in the chambers was controlled by Radiant version 2 software (Plexon).

##### Two-choice optogenetic self-stimulation task

Mice were first food restricted until they reached 85–90% of their free-feeding weight (∼7 d) to increase their motivation to produce instrumental behavioral responses. Then, in four consecutive daily sessions, mice were trained under a fixed ratio 1 [FR1 (one response produces the outcome)] schedule to instrumentally respond at a touch panel paired with the delivery of laser stimulation [S+ (30 s laser stimulation)] or a touch panel paired with delivery of no outcome (S–). The spatial (left/right) location of the S+ response panel was counterbalanced across mice. The outcome of each trial was followed by a 10 s intertrial interval (ITI). Each session lasted 60 min or until mice had completed 60 trials. The number of touch responses for the S+ and S– panels, as well as the latencies to make responses, were recorded in each session to assess the potential reinforcing effect of laser stimulation.

##### Two-choice task with optogenetic stimulation paired with a liquid reinforcer

Next, mice were trained to instrumentally respond at the same two response panels for delivery of a sucrose liquid reward (7 μl of 10% sucrose diluted in water); however, while a response at one panel (previously paired with laser delivery) was additionally paired with delivery of S+, a response at the other panel (S–) delivered just the liquid reward alone. Each outcome was followed by a 10 s ITI. The location of the S+ panel was counterbalanced across previous S+ and S– panels in the prior self-stimulation task. No main effect or interaction of previous panel location was observed, so data were grouped together.

Animals were trained on consecutive days using a previously described schedule of reinforcement with minor modifications (Robinson et al., 2014; Soares-Cunha et al., 2022), as follows: FR1 schedule for 5 d, FR4 schedule for 1 d, random-ratio 4 (RR4) schedule (a random number of responses between 1–4 produces the outcome) for 4 d, and finally RR6 for 4 d each. As previously, each session lasted 60 min or until 60 trials had been completed. The number of touch responses for the S+ (sucrose and laser) and the S– (sucrose alone), as well as the latencies to respond and collect the liquid rewards, was measured for each session.

### Open field test of motor activity

For bilateral stimulation tests, mice were placed in a gray cylindrical (diameter, 42 cm; height, 42 cm) open field apparatus. Patch cords were attached to bilateral fiber-optic cannulae and suspended above the animal so that they could freely move to all areas of the apparatus. Animals were allowed to freely explore the entire arena and habituate to the apparatus for 3 min before the start of testing. The test consisted of a 12-min-long session divided into four alternating 3 min trials during which bilateral laser stimulation was either OFF or ON (OFF-ON-OFF-ON), according to a previously described method (Tye et al., 2013). This protocol allows for observation not only of changes in locomotion following laser onset, but also for any prolonged effects of laser stimulation on locomotion during the second laser OFF period. During laser ON trials, photostimulation was delivered according to the protocol described in subsection Stereotaxic virus injection and optical cannula implantation. Total distance moved, velocity, and speed were automatically recorded by video tracking software (EthoVision XT 16, Noldus).

Unilateral stimulation was performed as described above, with the exception that only one patch cable was attached to either the contralateral or ipsilateral fiber-optic cannulae. Contralateral and ipsilateral tests were performed on consecutive days with the order randomized across mice. Body rotations (>180° turn) were automatically recorded using video tracking software (EthoVision XT 16, Noldus).

### Histologic analysis

After behavioral experiments were completed, mice were anesthetized with 90 mg/kg ketamine and 20 mg/kg xylazine, and transcardially perfused with 0.1 m PBS for 2 min followed by 4% paraformaldehyde (PFA; Nacalai Tesque) in 1× PBS for 5 min at a 10 ml/min flow rate. Brains were removed and postfixed overnight in 4% PFA, then placed in 7.5%, 15%, and 30% sucrose in 1× PBS solutions at 4°C until the brains sank in the solution at each stage. Brains were embedded and frozen completely in Optimal Cutting Temperature (O.C.T.) compound (Sakura Finetek). Then, the brain tissue was attached to a circular cryostat block and sectioned on a cryostat (model CM1860, Leica) into 40-μm-thick slices at −17 to −20°C. Coronal brain slices (40 μm) were stored in PBS solution at 4°C. For immunohistochemical staining, each brain section was treated with a blocking solution [5% bovine serum albumin (Nacalai Tesque) in 1× PBS] for 1 h at room temperature and washed three times in 1× PBS. After rinsing in 1× PBS, the slices were incubated in primary antibodies in 1× PBS with 0.3% Triton X (Nacalai Tesque; PBST) overnight at 4°C. For staining of EYFP, anti-green fluorescent protein rabbit IgG primary antibody (1:1000; Thermo Fisher Scientific) was used, for staining of tdTomato, anti-RFP antibody (1:1000; ABCAM) was used, and for staining of dopaminergic cells, anti-tyrosine hydroxylase (TH) antibody (1:500; EMD Millipore) was used. All brain sections were washed three times for 10 min in PBS, then stained with Alexa Fluor 488 or 555 goat anti-rabbit IgG secondary antibody (1:500; Thermo Fisher Scientific) in 1× PBST for 1 h at room temperature. After being washed again three times in 1× PBS for 10 min, the sections were mounted using Fluoroshield mounting medium containing DAPI (Abcam) and observed using a fluorescence microscope (model BZ-X800E All-In-One Fluorescence Microscope, KEYENCE).

### Statistical analyses

All experimental data were plotted as the mean ± SEM using Prism version 8.0 software (GraphPad Software) or SPSS software (IBM). Results from all statistical analyses are shown in Table 1. The rt-PP and open field test (OFT; bilateral stimulation) data were analyzed using two-way repeated-measures ANOVAs with virus (ChR2/ArchT/EYFP) as a between-subjects factor, laser (OFF/ON) as a within-subjects factor, and time spent in the laser-paired chamber minus time spent in the non-laser-paired chamber (for rt-PP), velocity (for OFT), or distance traveled (for OFT) as the dependent variable. The two-choice optogenetic operant tasks and unilateral stimulation OFT data were analyzed using three-way repeated-measures ANOVAs with virus (ChR2/ArchT/EYFP) as a between-subjects factor, panel (S+/S–), and session (day of training) or laser (ON/OFF) and turn direction (contralateral/ipsilateral rotation) as within-subjects factors, and responses (operant tasks) or turns (unilateral stimulation OFT) as the dependent variable. *Post hoc* Bonferroni’s multiple-comparisons tests were performed when ANOVA main effects or interactions were significant (*p* < 0.05). Mauchly’s sphericity test was used to assess the assumption of sphericity, and the Greenhouse–Geisser correction was applied where necessary (Mauchly’s test *p* < 0.05).

**Table 1:** Detailed statistical analyses of all experiments included in the manuscript

Figure		Description	*N*-number	Statistic	*p*-value	*Post hoc* test	*Post hoc p*-value
1	*E*	rt-PP, the preference time spent in the laser-paired chamber (in seconds)		2-way RM ANOVA		Bonferroni	
			D1-SNr/ChR2 10 mice, D1	Virus *F*_(2,24)_ = 20.77	*p* < 0.0001		ChR2, pretest vs laser test (day 1), ****p < 0.0001
							ChR2, pretest vs laser test (day 2), ****p < 0.0001
							ChR2, pretest vs laser test (day 3), ****p < 0.0001
			SNr/ArchT 8 mice, D1	Session *F*_(3,72)_ = 2.733	*p* < 0.05		ChR2 vs EYFP, laser test (day 1), ****p < 0.0001
							ChR2 vs ArchT, laser test (day 1), ****p < 0.0001
							ChR2 vs EYFP, laser test (day 2), ****p < 0.0001
			SNr/EYFP 9 mice	Virus × session *F*_(6,72)_ = 10.53	*p* < 0.0001		ChR2 vs ArchT, laser test (day 2), ***p < 0.001
							ChR2 vs EYFP, laser test (day 3), ****p < 0.0001
							ChR2 vs ArchT, laser test (day 3), ****p < 0.0001
2	*B*	Two-choice optogenetic stimulation, number of responses (days 1–4)	D1-SNr/ChR2 10 mice	3-way RM ANOVA		Bonferroni	
			D1-SNr/ArchT 8 mice	Virus *F*_(2,24)_ = 1.456	*p* = 0.253		ChR2, S+ vs S–, ***p < 0.001 (day 3)
			D1-SNr/EYFP 9 mice	Panel *F*_(1,24)_ = 10.050	*p* < 0.001		
				Session *F*_(3,72)_ = 0.372	*p* = 0.773		ChR2, S+ vs S–, ***p < 0.001 (day 4)
				Virus × panel *F*_(2,24)_ = 6.964	*p* < 0.001		ChR2 vs EYFP, S+, **p < 0.01 (day 3)
				Virus × session *F*_(6,72)_ = 1.413	*p* = 0.222		ChR2 vs EYFP, S+, **p < 0.01 (day 4)
				Panel × session *F*_(3,72)_ = 3.852	*p* < 0.05		ChR2 vs ArchT, S+, *p < 0.05 (day 3)
				Virus × panel × session *F*_(6,72)_ = 3.164	*p* < 0.01		ChR2 vs ArchT, S+, *p < 0.05 (day 4)
	*C*	Two-choice optogenetic stimulation, response latency (in seconds; days 1–4)		3-way RM ANOVA		N/A	N/A
				Virus *F*_(2,24)_ = 0.407	*p* = 0.670		
				Panel *F*_(1,24)_ = 0.025	*p* = 0.876		
				Session *F*_(3,72)_ = 1.534	*p* = 0.213		
				Virus × panel *F*_(2,24)_ = 0.438	*p* = 0.650		
				Virus × session *F*_(6,72)_ = 0.253	*p* = 0.957		
				Panel × session *F*_(3,72)_ = 0.307	*p* = 0.820		
				Virus × panel × session *F*_(6,72)_ = 0.889	*p* = 0.507		
	*E*	Two-choice optogenetic stimulation paired with a liquid reinforcer, number of responses (days 1–14)		3-way RM ANOVA		Bonferroni	ChR2, S+ vs S–, **p < 0.01 (day 2)
							ChR2, S+ vs S–, ****p < 0.0001 (day 3)
							ChR2, S+ vs S–, ****p < 0.0001 (day 4)
							ChR2, S+ vs S–, ****p < 0.0001 (day 5)
							ChR2, S+ vs S–, **p < 0.01 (day 6)
							ChR2, S+ vs S–, ***p < 0.001 (day 7)
							ChR2, S+ vs S–, **p < 0.01 (day 8)
							ChR2, S+ vs S–, **p < 0.01 (day 9)
							ChR2, S+ vs S–, ****p < 0.0001 (day 10)
							ChR2, S+ vs S–, **p < 0.01 (day 11)
							ChR2, S+ vs S–, ***p < 0.001 (day 12)
							ChR2, S+ vs S–, **p < 0.01 (day 13)
							ChR2, S+ vs S–, ****p < 0.0001 (day 14)
							ChR2 vs EYFP, S+, ***p < 0.001 (day 3)
							ChR2 vs EYFP, S+, ***p < 0.001 (day 4)
							ChR2 vs EYFP, S+, ***p < 0.001 (day 5)
							ChR2 vs EYFP, S+, **p < 0.01 (day 6)
							ChR2 vs EYFP, S+, *p < 0.05 (day 7)
							ChR2 vs EYFP, S+, *p < 0.05 (day 8)
							ChR2 vs EYFP, S+, **p < 0.01 (day 9)
							ChR2 vs EYFP, S+, **p < 0.01 (day 10)
							ChR2 vs EYFP, S+, *p < 0.05 (day 11)
							ChR2 vs EYFP, S+, *p < 0.05 (day 12)
							ChR2 vs EYFP, S+, *p < 0.05 (day 13)
							ChR2 vs EYFP, S+, **p < 0.01 (day 14)
							ChR2 vs ArchT, S+, *p < 0.05 (day 1)
							ChR2 vs ArchT, S+, ***p < 0.001 (day 2)
							ChR2 vs ArchT, S+, ****p < 0.0001 (day 3)
							ChR2 vs ArchT, S+, ****p < 0.0001 (day 4)
							ChR2 vs ArchT, S+, ****p < 0.0001 (day 5)
							ChR2 vs ArchT, S+, **p < 0.01 (day 6)
							ChR2 vs ArchT, S+, ***p < 0.001 (day 7)
				Virus *F*_(2,24)_ = 11.680	*p* < 0.0001		ChR2 vs ArchT, S+, **p < 0.01 (day 8)
				Panel *F*_(1,24)_ = 10.345	*p* < 0.01		ChR2 vs ArchT, S+, **p < 0.01 (day 9)
				Session *F*_(13,312)_ = 10.319	*p* < 0.0001		ChR2 vs ArchT, S+, ****p < 0.0001 (day 10)
				Virus × panel *F*_(2,24)_ = 37.016	*p* < 0.0001		ChR2 vs ArchT, S+, **p < 0.01 (day 11)
				Virus × session *F*_(26,312)_ = 1.849	*p* < 0.01		ChR2 vs ArchT, S+, **p < 0.01 (day 12)
				Panel × session *F*_(13,312)_ = 1.590	*p* = 0.087		ChR2 vs ArchT, S+, **p < 0.01 (day 13)
				Virus × panel × session *F*_(26,312)_ = 3.365	*p* < 0.0001		ChR2 vs ArchT, S+, ***p < 0.001 (day 14)
	*F*	Two-choice optogenetic stimulation paired with a liquid reinforcer, response latency (in seconds; days 1–14)		3-way RM ANOVA		N/A	ChR2 vs EYFP, *p < 0.05
				Virus *F*_(2,24)_ = 7.654	*p* < 0.01		
				Panel *F*_(1,24)_ = 0.590	*p* = 0.450		
				Session *F*_(13,312)_ = 0.835	*p* = 0.622		
				Virus × panel *F*_(2,24)_ = 0.104	*p* = 0.902		ChR2 vs ArchT, **p < 0.01
				Virus × session *F*_(26,312)_ = 1.064	*p* = 0.383		
				Panel × session *F*_(13,312)_ = 1.155	*p* = 0.312		
				Virus × panel × session *F*_(26,312)_ =1.147	*p* = 0.286		
	*G*	Two-choice optogenetic stimulation paired with a liquid reinforcer, reward correction latency (in seconds; days 1–14)		3-way RM ANOVA		Bonferroni	ChR2, S+ vs S-, *p < 0.05 (day 4)
				Virus *F*_(2,24)_ = 2.629	*p* = 0.093		ChR2, S+ vs S-, **p < 0.01 (day 9)
				Panel *F*_(1,24)_ = 6.248	*p* < 0.05		ChR2, S+ vs S-, *p < 0.05 (day 11)
				Session *F*_(13,312)_ = 1.039	*p* = 0.413		ChR2, S+ vs S-, ***p < 0.001 (day 12)
				Virus × panel *F*_(2,24)_ = 8.473	*p* < 0.01		ChR2, S+ vs S-, *p < 0.05 (day 13)
				Virus × session *F*_(26,312)_ = 0.772	*p* = 0.782		
				Panel × session *F*_(13,312)_ = 0.729	*p* = 0.735		
				Virus × panel × session *F*_(26,312)_ = 1.623	*p* < 0.05		

RM, Repeated-measures.

## Results

### Optogenetic stimulation of the NAc^D1-MSN^–SNr pathway drives reinforcement

**Figure 1.**
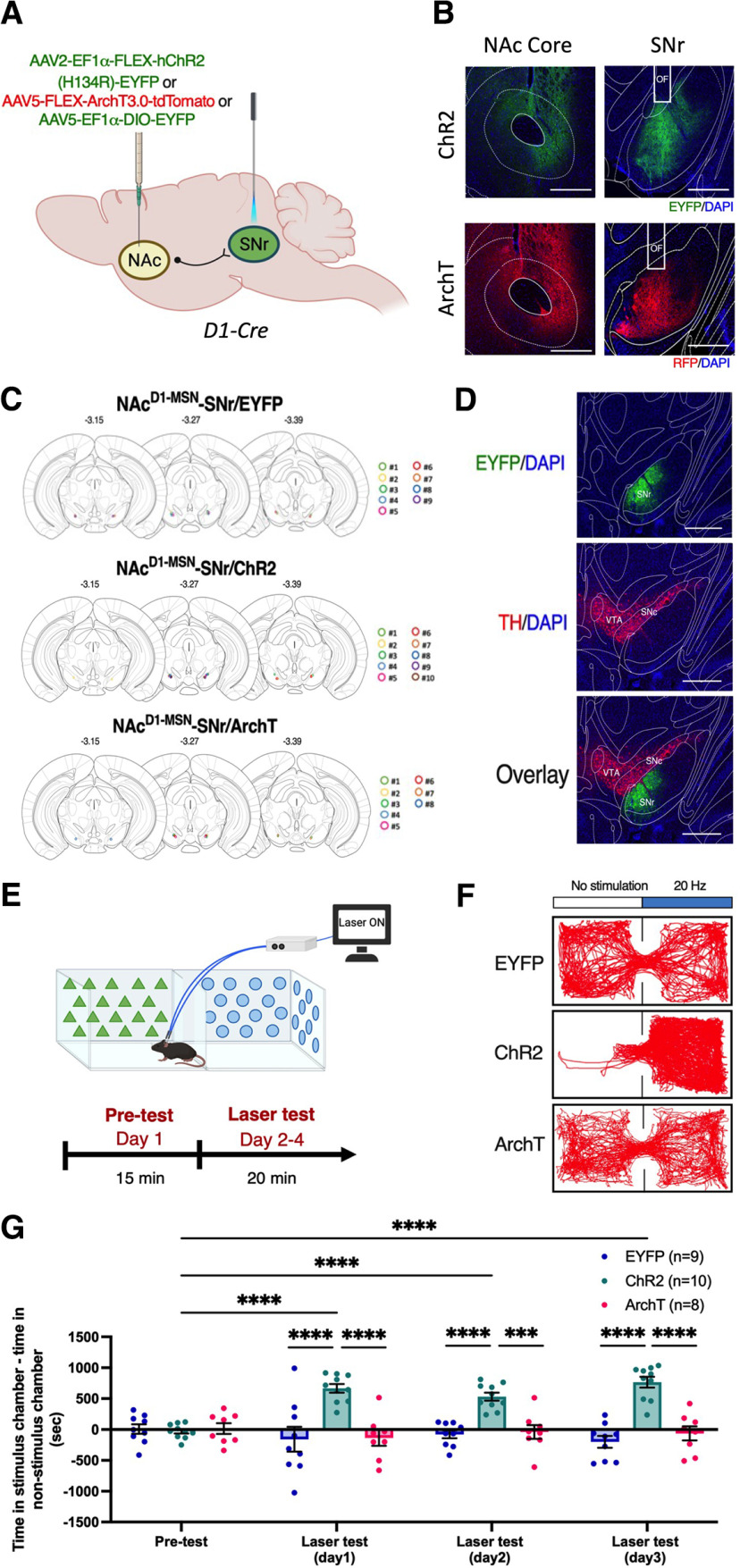
Optogenetic activation of the NAc^D1-MSN^–SNr pathway is reinforcing. ***A***, Schematic of viral infusion and optic fiber implantation sites in D1-Cre mice. ***B***, Representative coronal sections of ChR2-EYFP (top) and ArchT-tdTomato (bottom) expression in the NAc core (left) and SNr (right) of D1-Cre mice; nuclear marker (DAPI; blue); EYFP (green); RFP (red). OF, Optic fiber. Scale bar, 500 μm. ***C***, Representative coronal sections of optic fiber placements in the SNr in EYFP-expressing (*n* = 9), ChR2-expressing (*n* = 10), or ArchT-expressing (*n* = 8) mice. ***D***, Representative image of EYFP expression in the SNr (top), TH-positive dopaminergic neurons of SNc and VTA (middle), and an overlay of both (bottom). TH (red), EYFP (green), and DAPI (blue). Scale bar, 500 μm. ***E***, Schematic representation of the rt-PP assay and its experimental timeline. ***F***, Representative heatmaps of the time spent in each compartment during the laser test for three example D1-Cre mice expressing EYFP, ChR2, or ArchT, respectively. ***G***, Time spent in the laser-paired compartment minus time spent in the non-laser-paired chamber in D1-Cre mice expressing EYFP (*n* = 9), ChR2 (*n* = 10), or ArchT (*n* = 8) during pretest and laser test sessions. Data represent the mean ± SEM, *post hoc* Bonferroni comparisons. ***p <* *0.001, ****p <* *0.0001.

Given the established role of NAc D1-MSNs in reinforcement (Cole et al., 2018; Hikida et al., 2010; Lobo et al., 2010), we first investigated whether optogenetic activation or inhibition of the NAc^D1-MSN^–SNr pathway, via expression of the excitatory ChR2 or the inhibitory ArchT, respectively, could control reinforcement. Optic fibers were bilaterally implanted into the SNr of D1-Cre mice that had been microinjected with a Cre-dependent ChR2 [AAV2-Ef1a-FLEX-hChR2(H134R)-EYFP], ArchT (AAV5-FLEX-ArchT3.0-tdTomato), or EYFP control virus (AAV5-Ef1a-DIO-EYFP) to enable pathway-specific control of the activity of axon terminals [Fig. 1*A–C* (please note that experimental figures were created using BioRender.com)]. We further confirmed that virus expression at SNr terminal sites did not overlap with the dopaminergic neurons in the substantia nigra pars compacta (SNc) and the ventral tegmental area (VTA; Fig. 1*D*). In an rt-PP task, mice were first allowed to freely explore a two-chamber apparatus with differing contextual stimuli in each (triangles vs circles) for 15 min (Fig. 1*E*, Pretest). Then, on the next 3 consecutive days, mice were placed back in the apparatus for 20 min, which now produced laser stimulation via the optic fibers when mice entered into one of the two chambers (triangles vs circles, randomized between mice; Fig. 1*E*, Laser test). Mice expressing ChR2, but not ArchT or EYFP, were found to spend a significantly increased amount of time in the laser-paired chamber in the laser test sessions compared with the pretest session (Fig. 1*F*,*G*; significant virus × session interaction: *F*_(6,72)_ = 10.53, *p* < 0.0001), indicating a reinforcing effect of activation of the NAc^D1-MSN^–SNr pathway.

### Optogenetic activation of the NAc^D1-MSN^–SNr pathway is sufficient to support instrumental self-stimulation and augments instrumental responding for a liquid reinforcer

**Figure 2.**
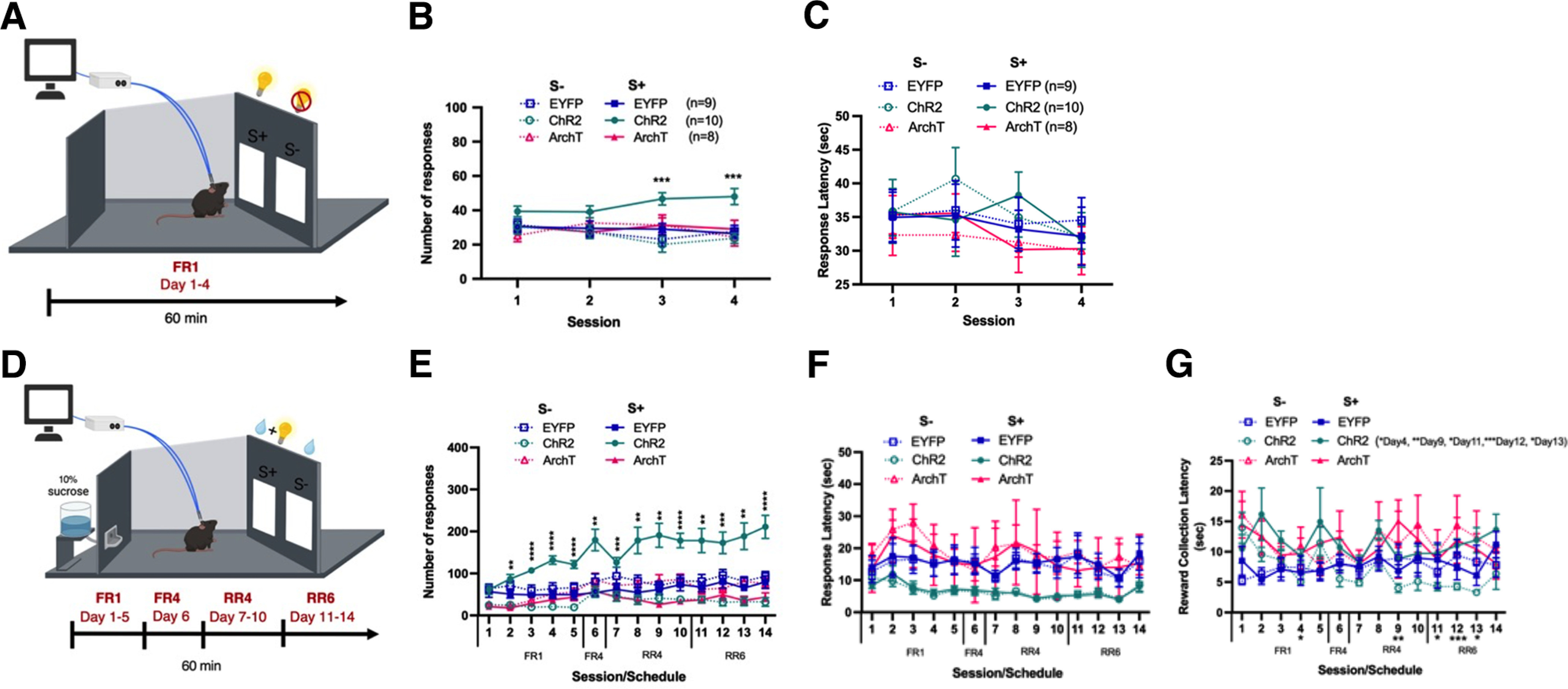
Optogenetic activation of the NAc^D1-MSN^–SNr pathway supports instrumental self-stimulation and increases instrumental responding for a liquid reinforcer. ***A***, Schematic representation and experimental timeline of the two-choice task in which mice instrumentally respond at two response panels under an FR1 schedule for either self-stimulation by the laser (S+) or no outcome (S–). ***B***, The total number of panel touches in mice receiving optogenetic activation (ChR2, *n* = 10) or inhibition (ArchT, *n* = 8) of the NAc^D1-MSN^–SNr pathway, as well as in control mice (EYFP, *n* = 9). ***C***, The latency to make a response at the S+ or S– panels in the two-choice optogenetic self-stimulation task under an FR1 schedule of reinforcement in mice expressing either EYFP (*n* = 9), ChR2 (*n* = 10), or ArchT (*n* = 8). ***D***, Schematic representation and experimental timeline of the two-choice task in which mice instrumentally respond at two response panels under an FR1-RR6 schedule for either a liquid reinforcer paired with laser delivery (S+) or the liquid reinforcer alone (S–). ***E***, The total number of panel touches in the FR1, FR4, RR4, and RR6 sessions of mice receiving optogenetic activation (ChR2) or inhibition (ArchT) of the NAc^D1-MSN^–SNr pathway (ChR2), as well as in control (EYFP) mice. ***F***, The latency to make a response at the S+ or S– panels in the two-choice laser and liquid reinforcer task under FR1, FR4, RR4, and RR6 schedules of reinforcement. ChR2 mice showed a reduced latency to make responses at S+ and S– panels across all sessions. ***G***, The latency to collect the liquid reward in the two-choice laser and liquid reinforcer task. ChR2 mice showed an increased latency to collect the liquid reward following an S+ response. Data represent the mean ± SEM. *Post hoc* Bonferroni comparisons: *p <* *0.05, **p <* *0.01, ***p <* *0.001, ****p <* *0.0001.

To further confirm the functional role of the NAc^D1-MSN^–SNr pathway in reinforcement, we next performed a two-choice schedule of operant self-stimulation. In four consecutive daily 60 min sessions in a touch-screen operant chamber, mice were trained under an FR1 schedule of reinforcement to produce a touch response at one of two response panels, one that resulted in a 30 s delivery of the laser via optic fibers (S+), and the other that resulted in no change (S–; Fig. 2*A*). Over the course of four test sessions, there was observed to be a significant increase in responding at the S+ panel compared with the S– panel in mice expressing ChR2, but not ArchT or EYFP (Fig. 2*B*; significant virus × panel × session interaction, *F*_(6,72)_ = 3.16, *p* < 0.01), indicating that activation of NAc D1-MSNs projecting to the SNr is sufficiently reinforcing to sustain instrumental responding. No difference in the latencies to respond at either of the response panels was observed among ChR2-expressing, ArchT-expressing, or EYFP-expressing mice across training sessions (Fig. 2*C*).

Next, to investigate whether, in addition to be being directly reinforcing, activation of this pathway could modulate the appetitiveness of a liquid reinforcer, we paired delivery of the laser with a response to earn a sucrose reward in a two-choice schedule of reinforcement (Fig. 2*D*). As previously, mice could choose to respond at one of two response panels. However, now a touch response at one randomly assigned panel delivered a sucrose reward alongside a 30 s laser delivery (S+), while a response at the other panel delivered the sucrose reward by itself (S–). Mice were trained on a previously described schedule of reinforcement with minor modifications (Robinson et al., 2014; Soares-Cunha et al., 2022), that proceeded from FR1 to RR6 (a random average of six responses needed to produce the outcome) over consecutive days. As with the previous self-stimulation task, it was revealed that ChR2-expressing mice responded a significantly greater number of times for the S+ panel compared with the S– panel, and that this effect gradually grew stronger across progressing schedules of reinforcement (Fig. 2*E*; significant virus × panel × session interaction: *F*_(26,312)_ = 3.37, *p *<* *0.0001). Analysis of response latencies indicated that ChR2-expressing mice were quicker to respond at both S+ and S– panels across all sessions compared with ArchT-expressing and EYFP-expressing mice (Fig. 2*F*; significant main effect of virus: *F*_(2,24)_ = 7.65, *p *<* *0.01). It is possible that the large reward (liquid reinforcer and laser) induced by responses at the S+ may have resulted in faster response times, an effect that was generalized onto S– responses too. Interestingly, the latency to collect the reward was significantly increased when mice expressing ChR2, but not ArchT or EYFP, responded at the S+ panel compared with the S– panel (Fig. 2*G*; significant virus × panel interaction: *F*_(2,24)_ = 8.47, *p *<* *0.01). We suggest that receipt of the rewarding laser stimulation may make mice less motivated to pick up the liquid reinforcer as they are already receiving a rewarding outcome.

Altogether, the findings of the rt-PP, two-choice self-stimulation, and laser paired with liquid reinforcer two-choice task indicate that activation of the NAc^D1-MSN^–SNr pathway is reinforcing and able to sustain and augment instrumental responding for the laser itself or a liquid reinforcer, respectively.

### Activation of the NAc^D1-MSN^–SNr pathway increases motor activity in an open field arena

**Figure 3.**
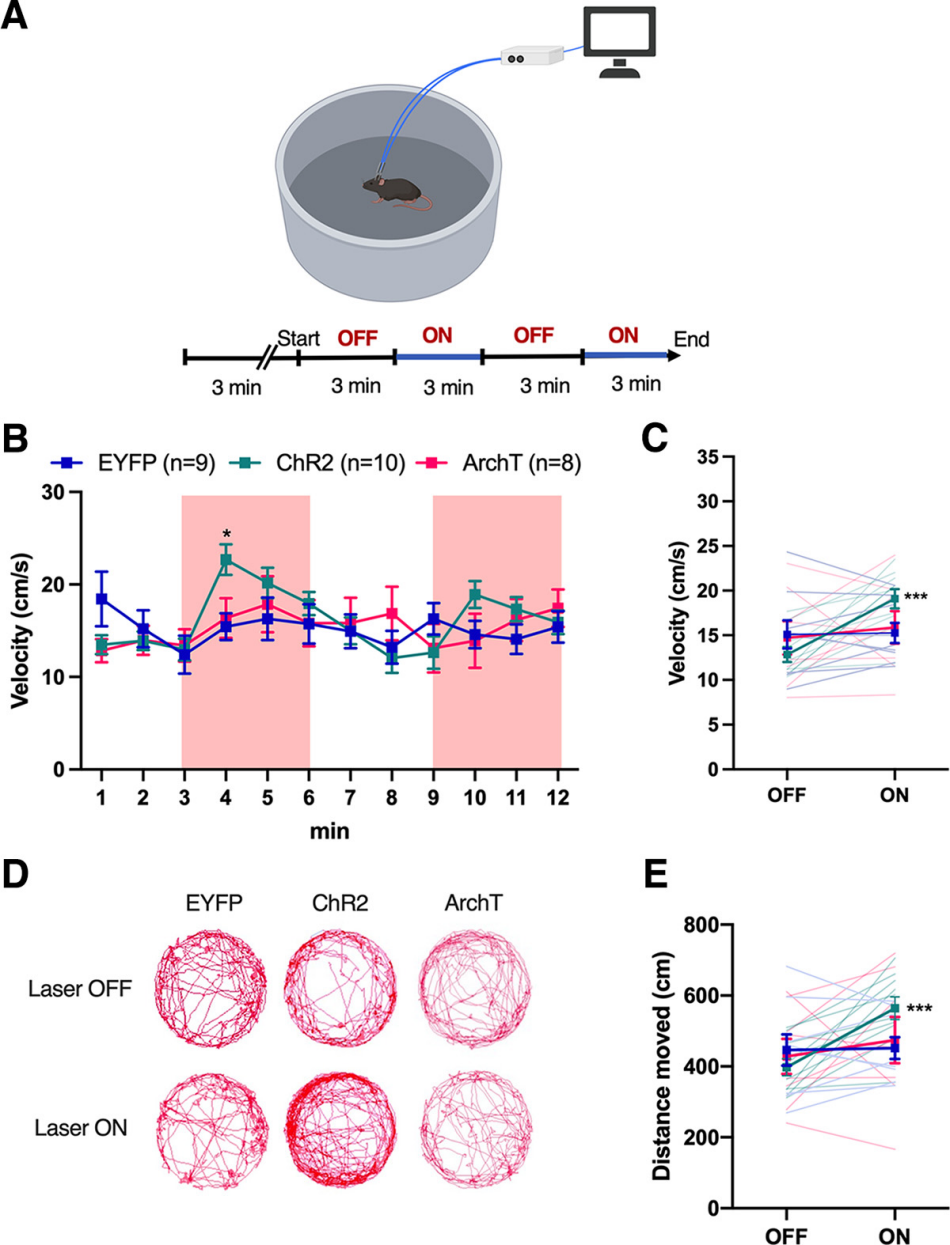
Bilateral activation of SNr-projecting NAc D1-MSNs increases locomotor activity. ***A***, Schematic representation and experimental timeline of the open field test in which mice move around a circular chamber during laser OFF and laser ON epochs. ***B***, The velocity of EYFP-expressing (*n* = 9), ChR2-expressing (*n* = 10), or ArchT-expressing (*n* = 8) mice during laser OFF and laser ON epochs across a 12 min session. Shaded areas indicate the time periods of laser delivery. ***C***, The average velocity during laser OFF and laser ON epochs in mice expressing ChR2, ArchT, or EYFP. ***D***, Representative image of the movement trace of an individual mouse in the open field arena during laser OFF (0–3 and 6–9 min) and laser ON (3–6 and 9–12 min) epochs. ***E***, The average total distance moved in during laser OFF and laser ON epochs in mice expressing ChR2, ArchT, or EYFP in the NAc^D1-MSN^–SNr pathway. Data represent the mean ± SEM. *Post hoc* Bonferroni comparisons: *p <* *0.05, ***p <* *0.001.

Next, as NAc core D1-MSN activation has previously been associated with augmented motor behavior (Dreher and Jackson, 1989; Plaznik et al., 1989; Zhu et al., 2016), we investigated the effect of excitation or inhibition of the NAc^D1-MSN^–SNr pathway on motor activity in an open field arena (Fig. 3*A*). Mice were first habituated to the apparatus for 3 min, then underwent four alternating 3 min laser OFF and laser ON epochs for a total of 12 min (Fig. 3*A*). Measurement of motor activity during the laser ON versus the laser OFF epochs revealed that laser stimulation of the axon terminals of NAc core D1-MSNs in the SNr resulted in a significant increase in velocity (Fig. 3*B*: significant virus × time period interaction: *F*_(22,264)_ = 1.96, *p* < 0.01; Fig. 3*C*: significant virus × laser interaction: *F*_(2,24)_ = 5.59, *p* < 0.05) and distance moved (Fig. 3*D*,*E*; significant virus × laser interaction: *F*_(2,24)_ = 4.27, *p* < 0.05) in ChR2-expressing mice, but no change in ArchT-expressing or EYFP-expressing mice.

**Figure 4.**
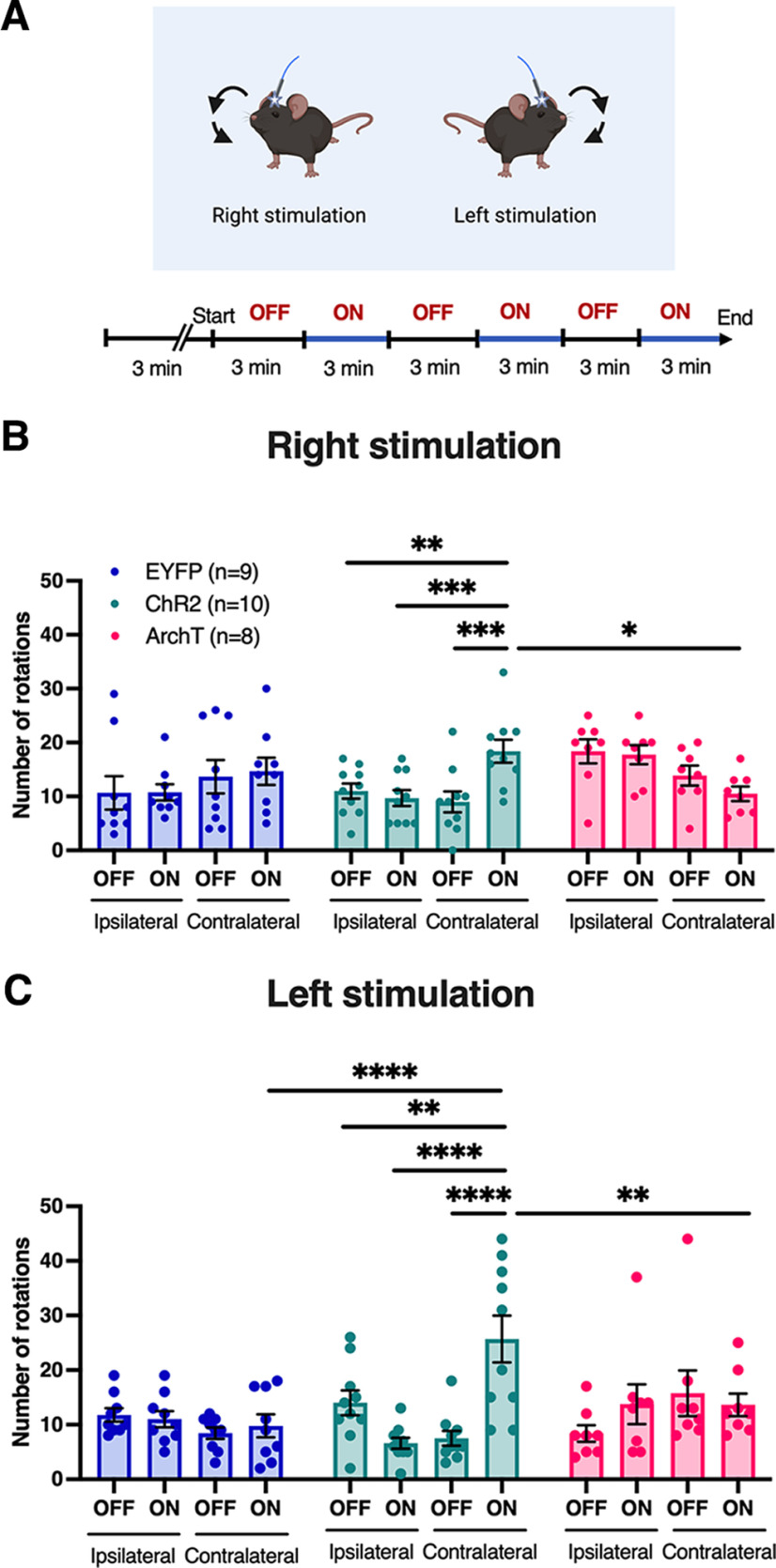
Unilateral activation of SNr-projecting NAc D1-MSNs induces contralateral rotations. ***A***, Schematic example of contralateral body rotations in the open field test following unilateral stimulation. ***B***, ***C***, Total number of ipsilateral and contralateral rotations during right stimulation (***B***) and left stimulation (***C***) in mice expressing EYFP (*n* = 9), ChR2 (*n* = 10), or ArchT (*n* = 8). Data represent the mean ± SEM. *Post hoc* Bonferroni comparisons: *p <* *0.05, **p <* *0.01, ***p <* *0.001, ****p <* *0.0001.

Finally, we next investigated whether unilateral stimulation of the NAc^D1-MSN^–SNr pathway is able to bias motor behavior toward the ipsilateral or contralateral direction to the stimulation side. Mice were habituated to the open field apparatus for 3 min then again underwent alternating 3 min laser OFF and laser ON epochs (Fig. 4*A*). As unilateral stimulation resulting in pronounced turning behavior rather than forward movement, the number of rotations to the ipsilateral or contralateral direction were measured during laser ON and laser OFF epochs. Laser stimulation of the NAc^D1-MSN^–SNr pathway resulted in a significant increase in contralateral turns to the stimulation side in mice expressing ChR2, but no change in those expressing ArchT or EYFP (Fig. 4*B*: significant virus × laser by turn direction interaction: *F*_(2,24)_ = 7.26, *p* < 0.01; Fig. 4*C*: significant virus × laser × turn direction interaction: *F*_(2,24)_ = 13.83, *p* < 0.0001).

Together, these findings indicate that activity in NAc core D1-MSNs projecting to the SNr is able to augment motor activity and control turning behavior, suggesting that this pathway may play an important role in motor control.

## Discussion

In this study, we found that activity in NAc core D1-MSNs projecting to the SNr is able to modulate both limbic and motor behaviors. Optogenetic activation of the axon terminals of NAc core D1-MSNs in the SNr was directly reinforcing and resulted in an increase in motor activity.

It was observed that mice showed a preference for a location paired with optogenetic activation of the NAc^D1-MSN^–SNr pathway and would instrumentally respond in an operant chamber to receive the optogenetic activation alone or optogenetic activation paired with a liquid reward. These findings support those of previous studies reporting that activation of NAc D1-MSNs is rewarding and able to augment the reinforcing effects of natural or drug rewards (Cole et al., 2018; Hikida et al., 2010; Lobo et al., 2010). Our findings also indicate that the reinforcing effects of cell body stimulation reported in these studies may occur via the projection from NAc D1-MSNs to the SNr. Indeed, while NAc core D1-MSNs also project to the VP, a recent study has indicated that optogenetic stimulation of this pathway in mice is aversive rather than reinforcing (Liu et al., 2022). Together these studies suggest a dissociation in the functional roles of NAc core D1-MSNs projecting to the SNr and VP in limbic control.

In our self-stimulation operant tasks, a laser stimulation duration of 30 s was used as it was estimated that this duration would provide sufficient stimulation for the mouse to quickly learn the contingency between the laser and the instrumental response. While it is difficult to directly compare this stimulation protocol to that of other rewarding stimuli including food and drugs or environmental stimuli conditioned to rewards, previous studies using *in vivo* calcium imaging have indicated that NAc D1-MSNs demonstrate prolonged activation to the delivery of sucrose rewards (excitation from baseline of >5 s for a single delivery of a 20 μl sucrose reward or a 20 mg sucrose pellet; Liu et al., 2022) or entry into a cocaine-associated environment (excitation from baseline that slowly reduces during the time in the cocaine-paired chamber; Calipari et al., 2016). These findings suggest that the 30 s duration used in our study may represent a similar neural response in D1-MSNs to that which might be expected by the prolonged consumption of a large reward. Interestingly, it has previously been reported that even brief (1 s) stimulation of D1-MSNs cell bodies in the NAc is sufficient to support optogenetic self-stimulation (Cole et al., 2018), although it should be taken into consideration that this study largely targeted D1-MSNs in the medial shell region of the NAc, which projects predominantly to dopaminergic neurons of the VTAs rather than to the SNr and therefore may produce a greater rewarding effect. Further investigation of the effect of different optogenetic stimulation protocols in different subregions of the NAc may help to further elucidate the role of NAc D1-MSNs in controlling reward-related behaviors.

Our finding that activation of the NAc^D1-MSN^–SNr pathway results in increased motor activity in an open field arena also support the findings of a recent study demonstrating that optogenetic stimulation of the axon terminals of NAc core D1-MSNs in the SNr resulted in increased activity in the M1 (Aoki et al., 2019). It is possible that, in our study, increases in velocity following bilateral stimulation and contralateral turning following unilateral stimulation are the result of a facilitation of M1 activity. Indeed, interestingly, it has recently been revealed that the limbic and motor basal ganglia loops may converge within the thalamus via overlapping patterns of innervation from medial and lateral SNr regions which themselves receive input from limbic-related nucleus accumbens and motor-related dorsolateral striatum regions, respectively (Aoki et al., 2019; Foster et al., 2021; Hunnicutt et al., 2014; Macpherson et al., 2021). Importantly, while motor activity was increased, our finding that instrumental responding in the self-stimulation experiments was largely selective to the stimulation-paired panel, rather than a general increase in responses at both panels, suggests that the findings of self-stimulation experiments are not simply the result of augmented motor activity.

Our studies revealed that excitation, but not inhibition, of the NAc^D1-MSN^–SNr pathway was able to alter reward-related and motor behaviors. These findings are in agreement with those of a previous study demonstrating that while chemicogenetic activation of NAc D1-MSNs increased the total distance run in a running wheel, chemicogenetic inhibition of NAc D1-MSNs did not affect motor behavior (Zhu et al., 2016). Interestingly, the same study revealed that chemicogenetic activation and inhibition of NAc D2-MSNs was able to reduce and increase wheel running, respectively. Together with our findings, these studies indicate that bidirectional control of motor behavior is possible via NAc D2-MSNs, which project to the VP, but not via NAc D1-MSNs projecting to the SNr. It remains to be investigated whether NAc D1-MSNs projecting to the VP may also contribute to motor control.

Interestingly, several psychiatric conditions associated with altered signaling in NAc D1-MSNs are characterized by abnormal limbic-related and motor-related behaviors. Indeed, locomotor sensitization, an augmentation of motor activity, is a common early behavioral adaptation to several addictive drugs, including methamphetamine, cocaine, ketamine, alcohol, nicotine, and opioids (Grahame et al., 2000; Ranaldi et al., 2009; Robinson and Berridge, 1993; Strong et al., 2017; Vezina, 2004), and has been reported to coincide with excessive dopamine release in the NAc core and increased activity in NAc D1-MSNs (di Chiara, 2002; van Zessen et al., 2021). Oppositely, motor retardation is a common symptom of both major depression and the depressed phase of bipolar disorder in humans (Buyukdura et al., 2011; Caligiuri and Ellwanger, 2000) and is also reported in chronic social defeat mouse models of depression (Huang et al., 2013; Ota et al., 2018). Accordingly, chronic social defeat in mice is associated with reduced NAc dopamine release and reduced excitatory synaptic input onto NAc D1-MSNs (Francis et al., 2015), an effect that could be predicted to reduce signaling through the NAc^D1-MSN^–SNr pathway. While evidence of a common etiology in the limbic and motor symptoms of psychiatric disorders, including depression and substance abuse, is yet to be established, our data suggest the NAc^D1-MSN^–SNr pathway may present an attractive target for future investigation.

Overall, our findings revealed that activity in NAc core D1-MSNs projecting to the SNr was able to influence both reinforcement and motor behavior, indicating a multifunctional role of this pathway. These studies also provide further evidence in support of the convergence of limbic and motor basal ganglia circuits, and may help to provide a plausible neural circuit contributing to the frequent cooccurrence of limbic and motor symptoms in psychiatric conditions including depression and substance abuse.

## Synthesis

Reviewing Editor: Erica Glasper, The Ohio State University Decisions are customarily a result of the Reviewing Editor and the peer reviewers coming together and discussing their recommendations until a consensus is reached. When revisions are invited, a fact-based synthesis statement explaining their decision and outlining what is needed to prepare a revision will be listed below. The following reviewer(s) agreed to reveal their identity: Kurt Fraser.

Overview: This well-written manuscript uses cell-type specific optogenetics to examine the function of the projection of D1 dopaminergic receptors on medium spiny neurons (D1 MSNs) in the nucleus accumbens core to the nucleus accumbens in male mice. The authors demonstrate that not only does stimulation elicit locomotion, but it also supports primary reinforcement and augments the reinforcing value of a real-world natural reinforcer. The experiments were well-designed, and the analyses appear appropriate. The findings reported in this manuscript are likely to advance the field. There are, however, some concerns that if sufficiently answered could increase clarity and interpretation of the data. When required for clarity, specific reviewers’ comments have been italicized and quoted.

Major Concerns

1. The use of only male mice should be mentioned throughout the manuscript, including the title, abstract, and significance statement.

2. Please justify the use of a 30s laser stimulation and the 3 min of stimulation for the locomotor activity testing. Please discuss the extent to which theses parameters reflect activity of D1 MSNs. Particular attention to the ICSS and reward augmentation task should be addressed.

3. The observation of selective terminals in the SNr was surprising. Prior researchers “suggest that these neurons also project to the VTA and SNc. I was wondering why the expression appears highly specific to the SNr in this case (e.g., Yang H et al, 2018). As a result I am concern about the specificity of these manipulations as solely reflecting Nac->SNr connectivity and not the complex effects of the stimulation on VTA and SNc connections as well.”

4. “It seems to me that the study would be more conclusive if they showed the effect of inactivation of the D1-MSNs projecting to SNr or show the effect if the motor effect occurs in the presence of D1 antagonism.”

Minor Concerns

1. Line 76 - Please correct the typo in “activation optogenetic activation”.

2. Please explain why mice turn more when they are unilaterally stimulated on the left side.

3. Please number all figures so that they are easier to recognize.

## Author Response

Authors Comment:

We thank the reviewers for their helpful comments. We have now made considerable corrections to the manuscript that we hope address all the reviewers’ comments. We have marked changes within the manuscript in red.

Reviewer’s Comment:

Major Concern

1. The use of only male mice should be mentioned throughout the manuscript, including the title, abstract, and significance statement.

Authors Reply:

We thank the reviewer for their comment.

We have now amended the title, abstract, and significance statement to clarify that only male mice were used. (lines 1-2, 7-10, and 19-21).

Reviewer’s Comment:

Major Concern

2. Please justify the use of a 30s laser stimulation and the 3 min of stimulation for the locomotor activity testing. Please discuss the extent to which theses parameters reflect activity of D1 MSNs. Particular attention to the ICSS and reward augmentation task should be addressed.

Authors Reply:

We thank the reviewer for their comment.

The 30 sec laser stimulation as chosen as we estimated that this would provide sufficient stimulus for the mouse to quickly learn the contingency between the laser and the instrumental response and to demonstrate the appropriate behavior (reinforcement or avoidance). While the precise physiological response dynamics of NAc D1-MSNs to rewards is still unclear, experiments using fiber photometry recording of NAc D1-MSNs have indicated that these neurons display prolonged excitation to the delivery of rewarding stimuli, including sucrose (excitation of over 5 sec for a single delivery of a 20μl sucrose reward or a 20mg sucrose pellet, Liu et al., 2021, Cell Research) or presence within a cocaine-associated location (excitation that slowly reduces during the time in the cocaine-paired chamber, Calipari et al., 2016, PNAS). It is difficult to directly compare our laser stimulation protocol with food/drug stimuli, but we would speculate that the 30 sec laser stimulation in the present study would represent a large reward to the mouse. The stimulation protocol for the locomotor activity testing was based on a previous protocol used in Tye et al., 2013, Nature. The 3-min alternating Laser-ON and Laser-OFF periods allow for observation not only of the speed of changes in locomotion following laser onset, but also for any prolonged effects of laser stimulation on locomotion during the second laser-Off period.

We have now added greater detail and discussion of the laser stimulation protocols to the methods and discussion sections (lines 190-192, and 355-374).

Reviewer’s Comment:

Major Concern

3. The observation of selective terminals in the SNr was surprising. Prior researchers suggest 2 that these neurons also project to the VTA and SNc. I was wondering why the expression appears highly specific to the SNr in this case (e.g., Yang H et al, 2018). As a result I am concern about the specificity of these manipulations as solely reflecting Nac->SNr connectivity and not the complex effects of the stimulation on VTA and SNc connections as well.”

Author’s Reply:

We thank the reviewer for their comment.

Previous studies using anterograde tracing have indicated that the NAc core region (specifically D1-MSNs of the NAc core) projects primarily to the medial SNr, with minimal projection to the SNc and VTA (Heimer et al., 1991, Neuroscience; Stefanik et al., 2013. J.Neurosci; Smith et al., 2013, Curr Opin Neurobiol; Aoki et al., 2019, eLife). In contrast, D1- MSNs in the medial and lateral NAc shell regions (including those targeted in Yang H et al., 2018) project primarily to the medial and ventral VTA, respectively. We agree with the reviewer that spillover stimulation to the VTA and SNc could potentially confound the results of our study and were therefore careful to confirm using immunohistochemical analysis that NAc core D1-MSN axon terminals were restricted to the SNr and not VTA/SNc regions (see Supplementary figure S1D).

Reviewer’s Comment:

Major Concern

4. It seems to me that the study would be more conclusive if they showed the effect of inactivation of the D1-MSNs projecting to SNr or show the effect if the motor effect occurs in the presence of D1 antagonism.

Author’s Reply:

We thank the reviewers for their comments.

We agree with the reviewers that inhibition studies would also be useful to support the conclusions of our study and were in the process of performing such studies when the manuscript was submitted. We have now completed and added these experiments to the manuscript. Interestingly, no effect was observed of optogenetic inhibition of NAc D1-MSNs on reward-related or motor behaviors, indicating that this pathway does not bidirectionally control these functions. We have now updated the figures (fig. 1-6), abstract (lines 26-27), introduction (lines 82-83), methods (105-113, 122-125, 216-220, 228-232, 231-240), and results (248-256, 266-267, 277-281, 293-304, 313-316, 318-323, 330-334), and have included a discussion of the results within the discussion section (lines 390-399).

Reviewer’s Comment

Minor Concern

1. Line 76 - Please correct the typo in “activation optogenetic activation”.

Author’s Reply:

We thank the reviewers for their comment. We have now corrected this typo.

Reviewer’s Comment

Minor Concern

2. Please explain why mice turn more when they are unilaterally stimulated on the left side.

3

Author’s Reply:

We thank the reviewer for their comment. In response to the reviewer’s comment, we performed a 4-way repeated measures ANOVA and found a significant Direction (right/left) * Side (ipsilateral/contralateral) * Laser (on/off) * virus (EYFP/ChR2/ArchT) interaction (F(2,24)=3.99, p < 0.05; Bonferroni post hoc, right contralateral ChR2 vs left contralateral ChR2, p < 0.05), indicating that as the reviewer points out, there appears to be significantly increased contralateral turning in ChR2 mice when they were stimulated on the left side compared to when they were stimulated on the right side. We first speculated that such differences may arise from differences in virus expression or optic fiber tip placement in the SNr between the left and right sides; however, histological analysis of ChR2 mice did not reveal any such noticeable differences. Interestingly, a similar bias in turning between stimulation sides has previously been reported in a study using a similar unilateral stimulation task, albeit to the opposite contralateral side (right rather than left) and in a different part of the brain (subthalamic nucleus) (Guillaumin et al., 2021, Brain Research).

The underlying cause of this bias is yet to be determined, but potentially there may be hemispheric differences between control of motor behavior by the right and left NAcD1-MSN-SNr pathways.

Reviewer’s Comment

Minor Concern

3. Please number all figures so that they are easier to recognize.

Author’s Reply:

We have now numbered all figures so that they can be easily recognized.
